# Correlation of Triglyceride-Glucose Index and the Presence of Microvascular Complications of Diabetes

**DOI:** 10.7759/cureus.90363

**Published:** 2025-08-18

**Authors:** Chelsea S Miranda, Pinto VJ, Chris Miranda

**Affiliations:** 1 Internal Medicine, Father Muller Medical College, Mangalore, IND; 2 Respiratory Medicine, King George Hospital, Ilford, GBR

**Keywords:** diabetic, diabetic nephropathy (dn), diabetic peripheral neuropathy (dpn), diabetic retinopathy, triglyceride-glucose index, types 2 diabetes

## Abstract

Background

In recent decades, there has been a substantial rise in the global burden of diabetes and the complications associated with it. Among the numerous complications, the most prevalent are the microvascular ones, namely, diabetic retinopathy (DR), neuropathy, and nephropathy. These conditions contribute considerably to both morbidity and mortality in individuals with diabetes. Early identification and management of microvascular complications not only help in mitigating their progression but may also play a role in preventing subsequent macrovascular events, such as ischemic heart disease and cerebrovascular accidents. This underscores the importance of identifying reliable, accessible markers that can indicate the presence or potential risk of developing such complications. The triglyceride-glucose (TyG) index, derived from fasting blood glucose and triglyceride levels, is a simple and routinely obtainable measure that has gained attention for its possible role in this context. This study aims to evaluate the association between the TyG index and the occurrence of microvascular complications in individuals with type 2 diabetes mellitus (T2DM).

Objectives

This study aimed to estimate the TyG index in T2DM patients and to correlate the relationship of the TyG index with the presence of DR, nephropathy, and neuropathy.

Materials and methods

A cross-sectional study was conducted on 125 patients who met the inclusion and exclusion criteria. A detailed history was taken, and a thorough clinical examination was performed. Fasting triglyceride and fasting blood sugar levels were measured to calculate the TyG index. Urine microalbumin was measured for the evaluation of diabetic nephropathy. Peripheral neuropathy was evaluated by testing sensory perceptions of light touch, temperature, pain, and vibration sense, along with the presence or absence of ankle jerk. The fundus was visualized using an ophthalmoscope for DR.

Result

A total of 125 subjects were studied, which included 71 (56.8%) males and 54 (43.2%) females. The average age of the participants was 61.23 years, with the age ranging from 25 to 90 years. The mean duration of diabetes in this study was 7.8 years. Out of 125 participants, 51 (40.8%) had DR, 62 (49.6%) had microalbuminuria, and 66 (52.8%) had peripheral neuropathy. The mean value of TyG was 9.2449. The TyG index showed significant correlation with DR, microalbuminuria, and peripheral neuropathy. It was also found that there was a significant correlation between the duration of diabetes and the development of diabetic neuropathy.

Conclusion

The TyG index correlated positively with DR, peripheral neuropathy, and microalbuminuria.

## Introduction

Diabetes mellitus (DM) is a chronic metabolic disorder characterized by persistent hyperglycemia due to impaired insulin secretion, defective insulin action, or both. Prolonged elevation in blood glucose levels can result in multiorgan damage, primarily affecting the cardiovascular system, kidneys, retina, and nervous system. According to the latest WHO data of 2022, an estimated 830 million people worldwide have diabetes, with 1.6 million deaths annually attributed to the disease [1]. India, often referred to as the “diabetes capital” of the Southeast Asian region, has the highest prevalence of diabetes, with 74 million affected individuals. The age-adjusted comparative prevalence of type 2 diabetes mellitus (T2DM) in India is 9.8% among adults between 18 and 99 years. Alarmingly, diabetes accounts for 50.7% of premature mortality (ages 20-79) in the country [2].

Diabetes imposes a significant healthcare burden due to its complications, and these are broadly categorized into macrovascular and microvascular categories. Macrovascular complications involve larger arteries, increasing the risk of cardiovascular and cerebrovascular diseases. Microvascular complications affect small blood vessels and include conditions such as retinopathy, nephropathy, and neuropathy. Microvascular complications are said to develop in tissues where glucose uptake is insulin-independent, such as the vascular endothelium, retina, and kidneys. These tissues are directly exposed to blood glucose levels, leading to endothelial damage through glucose-mediated toxicity, oxidative stress caused by superoxide overproduction, and the accumulation of sorbitol and advanced glycation end-products [3], all of which contribute to disease progression and complications. Microvascular complications are also early indicators of cardiovascular and cerebrovascular morbidity and mortality in diabetic patients. Their presence signals an increased risk of systemic vascular damage, making early detection essential for timely intervention. Identifying these complications at an early stage enables proactive management strategies to prevent further progression and reduce the overall burden of diabetes-related complications.

Several epidemiological studies have highlighted the burden of microvascular complications in India. As per the Chennai Urban Rural Epidemiology Study (CURES Study), the prevalence of diabetic retinopathy (DR) is at 17.6% [4], while the prevalence of diabetic nephropathy ranges from 0.9% to 62.3% [5] and diabetic neuropathy from 10.5% to 44.9% [6]. It is found that diabetic nephropathy remains the leading cause of end-stage renal disease (ESRD) worldwide, with 20% of T2DM patients progressing to ESRD during their lifetime [7], while approximately 80% of diabetics develop DR, which is one of the leading global causes of irreversible blindness and the main cause of blindness in adults of working age [8]. These statistics highlight the need for improved screening and management strategies. Clinical trials, including the UK Prospective Diabetes Study (UKPDS) [9] and the Diabetes Control and Complications Trial (DCCT) [10], have demonstrated a strong correlation between poor glycemic control and microvascular complications. Insulin resistance (IR) plays a central role in this process, making its early identification essential for preventing disease progression. The gold standard for measuring IR is the euglycemic-hyperinsulinemic clamp [11]. However, this method is time-consuming and costly, limiting its clinical utility. A simpler and frequently used alternative is the Homeostasis Model Assessment for Insulin Resistance (HOMA-IR) [12], calculated as follows:

$\text{HOMA-IR}= \frac{\text{Fasting insulin(U/ml)}\times \text{Fasting glucose(mmol/L)}}{22.5}$

Despite its utility, HOMA-IR relies on plasma insulin or C-peptide assays, which are expensive and not widely available. It is well established that triglyceride and glucose levels play pivotal roles in cardiovascular and metabolic risk assessment. Triglyceride-rich lipoproteins (TGRLs), such as very-low-density lipoproteins (VLDL) and chylomicrons, have shown conflicting associations with cardiovascular outcomes. Large-scale epidemiological studies, such as the Copenhagen City Heart Study [13], have reported an elevated cardiovascular risk with higher triglyceride levels. However, interventional trials aimed at lowering triglyceride levels, such as the FIELD trial [14], have not consistently demonstrated reductions in cardiovascular events.

On a theoretical level, hepatic de novo lipogenesis, predominantly stimulated by excess carbohydrate intake, is linked to selective hepatic IR and hyperinsulinemia, thereby promoting increased VLDL secretion and the development of hypertriglyceridemia [15]. In this context, triglycerides may serve as surrogate markers of IR, a metabolic state associated with both macrovascular disease and microvascular complications of diabetes. Furthermore, assessing triglyceride and glucose levels together may enhance the detection of individuals at risk for atherosclerotic cardiovascular disease and end-organ damage, even in the absence of overt dyslipidemia [16].

The triglyceride-glucose (TyG) index [17] is an emerging, cost-effective alternative for assessing IR. It is calculated as follows:

$\text{TyG index}= \ln\frac{\text{Fasting triglyceride(mg/dl)}\times\text{Fasting glucose(mg/dl)} }{2}$

Studies suggest that the TyG index correlates strongly with IR and microangiopathy, making it a promising tool for clinical practice. However, limited data exist on its association with specific microvascular complications, such as DR, nephropathy, and neuropathy. Given the rising burden of diabetes and its complications, there is a critical need for simple yet effective markers of IR and microvascular risk stratification. The TyG index presents a promising alternative to traditional methods, warranting further research to validate its clinical application. Future studies should focus on establishing threshold values for the TyG index and exploring its predictive utility in preventing diabetes-related complications.

## Materials and methods

Study design and source of data

A cross-sectional study was carried out on patients with T2DM who presented to Father Muller Medical College Hospital, Mangalore, from January 2020 to June 2021 for a period of 18 months. Patients fulfilling the inclusion criteria were evaluated for microvascular complications, and their TyG index was calculated.

Selection criteria

Inclusion Criteria

All diagnosed cases of T2DM as per the American Diabetes Association (ADA) criteria [18] were included in the study, irrespective of their treatment status. *Exclusion Criteria*

Diabetics with chronic kidney disease (CKD) who are undergoing dialysis or have an estimated glomerular filtration rate (eGFR) of <15 mL/min/1.73 m^2^, patients presenting with acute complications of T2DM (diabetic ketoacidosis and hyperglycemic hyperosmolar state), pregnant women, and individuals with any febrile illness, severe heart failure, and a family history of hypertriglyceridemia were excluded from the study.

Sample size calculation

Considering an 8.9% prevalence rate of diabetes in India as per the International Diabetes Federation, March 2020, and an allowable error of 5%, a sample size of 125 was obtained using the Cochran’s sample size formula.

$n=Z^{2}\alpha\times \frac{p\left( 1-p \right)}{e^{2}}$

where p is the prevalence rate, taken as 8.9% according to the International Diabetes Federation, March 2020, e is the allowable error of 5%, and Zα is the relative deviate.

Statistical analysis 

The studied patients were stratified into four groups according to the TyG index quartile. The data were expressed as percentages for categorical variables or mean ± SD for continuous variables and were analyzed using IBM SPSS Statistics for Windows, Version 20 (Released 2011; IBM Corp., Armonk, New York, United States). The results were interpreted using mean, standard deviation, chi-square test, independent t-test, and one-way analysis of variance (ANOVA) test.

Sampling method and data collection

This study employed simple random sampling to select diabetic patients for inclusion. A detailed medical history was obtained from all participants, followed by a comprehensive clinical examination. Urine microalbumin levels and serum triglyceride concentrations were measured using the Roche Diagnostics Cobas C501 clinical chemistry autoanalyzer. Peripheral neuropathy was assessed by evaluating sensory perception through multiple modalities: light touch using a 10 g monofilament, pain sensation using pinprick, vibration sense using a 128 Hz tuning fork, temperature discrimination, and the ankle jerk reflex. Screening for DR was conducted by an ophthalmologist through direct fundus examination using a direct ophthalmoscope.

## Results

A total of 125 patients were included in the study, of which 71 (56.8%) were males with an average body mass index (BMI) of 24.8 kg/m², and 54 were females (43.2%) with an average BMI of 25.8 kg/m². Most of the patients were aged between 61 and 70 years, and the average duration of illness was 7.8 years. A history of microvascular complications was given by 57 patients, of whom 28 had paresthesias, 24 had a history of diabetic foot, and two of them gave a history that was suggestive of autonomic dysfunction. Macrovascular complications were seen in 44 patients, of whom 12 had a previous stroke, 29 had coronary artery disease, and two had peripheral artery occlusive disease. 

The TyG index was stratified into four quartiles (TyG index-Q) based on the distribution of values within the study population: quartile 1 (<8.85), quartile 2 (8.85-9.10), quartile 3 (9.10-9.60), and quartile 4 (>9.60). Quartile cut-off points were determined by arranging all participants’ TyG index values in ascending order and dividing them into four equal groups, each containing approximately 25% of the sample. This stratification allowed for trend analysis across increasing TyG index levels. Table 1 shows baseline patient characteristics in the four TyG index-Q.

**Table 1:** Baseline characteristics in the TyG index quartiles TGI: triglyceride-glucose index; TyG: triglyceride-glucose; OHA: oral hypoglycemic agent; HTN: hypertension; CVA: cerebrovascular accident; CAD: coronary artery disease; PAOD: peripheral artery occlusive disease; OSA: obstructive sleep apnea

	Categories	N	TGI quartiles				Chi-square	p-value
			Q1 (<8.85) (N (%))	Q2 (8.85-9.1) (N (%))	Q3 (9.1-9.6) (N (%))	Q4 (>9.6) (N (%))		
Age	<30	1	0 (0)	0 (0)	0 (0)	1 (3.1)	19.69	0.351
	31-40	3	0 (0)	1 (3.2)	1 (3.2)	1 (3.1)		
	41-50	20	5 (16.1)	2 (6.5)	5 (16.1)	8 (25)		
	51-60	35	7 (22.6)	10 (32.3)	11 (35.5)	7 (21.9)		
	61-70	41	11 (35.5)	10 (32.3)	9 (29)	11 (34.4)		
	71-80	18	3 (9.7)	7 (22.6)	4 (12.9)	4 (12.5)		
	>80	7	5 (16.1)	1 (3.2)	1 (3.2)	0 (0)		
Sex	Female	54	14 (45.2)	14 (45.2)	14 (45.2)	12 (37.5)	0.57	0.903
	Male	71	17 (54.8)	17 (54.8)	17 (54.8)	20 (62.5)		
Family history	No	54	17 (54.8)	14 (45.2)	14 (45.2)	9 (28.1)	4.772	0.189
	Yes	71	14 (45.2)	17 (54.8)	17 (54.8)	23 (71.9)		
Diabetes mellitus treatment modality	nil	10	2 (6.5)	2 (6.5)	2 (6.5)	4 (12.5)	23.405	0.024
	Insulin	10	2 (6.5)	3 (9.7)	1 (3.2)	4 (12.5)		
	Lifestyle modification	3	2 (6.5)	0 (0)	0 (0)	1 (3.1)		
	OHA	85	25 (80.6)	23 (74.2)	24 (77.4)	13 (40.6)		
	OHA & insulin	17	0 (0)	3 (9.7)	4 (12.9)	10 (31.2)		
Other comorbidities								
HTN	No	67	17 (54.8)	18 (58.1)	14 (45.2)	18 (56.2)	1.246	0.742
	Yes	58	14 (45.2)	13 (41.9)	17 (54.8)	14 (43.8)		
CVA	No	115	31 (100)	28 (90.3)	28 (90.3)	28 (87.5)	3.813	0.282
	Yes	10	0 (0)	3 (9.7)	3 (9.7)	4 (12.5)		
CAD	No	107	28 (90.3)	27 (87.1)	23 (74.2)	29 (90.6)	4.545	0.208
	Yes	18	3 (9.7)	4 (12.9)	8 (25.8)	3 (9.4)		
PAOD	No	124	31 (100)	31 (100)	31 (100)	31 (96.9)	2.93	0.403
	Yes	1	0 (0)	0 (0)	0 (0)	1 (3.1)		
OSA	No	124	30 (96.8)	31 (100)	31 (100)	32 (100)	3.057	0.383
	Yes	1	1 (3.2)	0 (0)	0 (0)	0 (0)		
History suggestive of microvascular complications		71	25 (80.6)	19 (61.3)	15 (48.4)	12 (37.5)	13.19	0.004
	Yes	54	6 (19.4)	12 (38.7)	16 (51.6)	20 (62.5)		
History suggestive of macrovascular complications	No	81	24 (77.4)	22 (71)	16 (51.6)	19 (59.4)	5.458	0.141
	Yes	44	7 (22.6)	9 (29)	15 (48.4)	13 (40.6)		
Evaluation for microvascular complications								
Peripheral neuropathy	No	59	24 (77.4)	15 (48.4)	12 (38.7)	8 (25)	18.602	<0.001
	Yes	66	7 (22.6)	16 (51.6)	19 (61.3)	24 (75)		
Urine microalbumin (mg/dL)	No	63	28 (90.3)	21 (67.7)	7 (22.6)	7 (21.9)	43.507	<0.001
	Yes	62	3 (9.7)	10 (32.3)	24 (77.4)	25 (78.1)		
Fundoscopy	No Retinopathy	74	26 (83.9)	16 (51.6)	18 (58.1)	14 (43.8)	21.376	0.045
	Mild NPDR	26	4 (12.9)	10 (32.3)	3 (9.7)	9 (28.1)		
	Moderate NPDR	17	0 (0)	5 (16.1)	6 (19.4)	6 (18.8)		
	Severe NPDR	2	0 (0)	0 (0)	1 (3.2)	1 (3.1)		
	PDR	6	1 (3.2)	0 (0)	3 (9.7)	2 (6.2)		
OHA/insulin	nil	10	2 (6.5)	2 (6.5)	2 (6.5)	4 (12.5)	23.405	0.024
	Insulin	10	2 (6.5)	3 (9.7)	1 (3.2)	4 (12.5)		
	Lifestyle modification	3	2 (6.5)	0 (0)	0 (0)	1 (3.1)		
	OHA	85	25 (80.6)	23 (74.2)	24 (77.4)	13 (40.6)		
	OHA & insulin	17	0 (0)	3 (9.7)	4 (12.9)	10 (31.2)		

**Figure 1:**
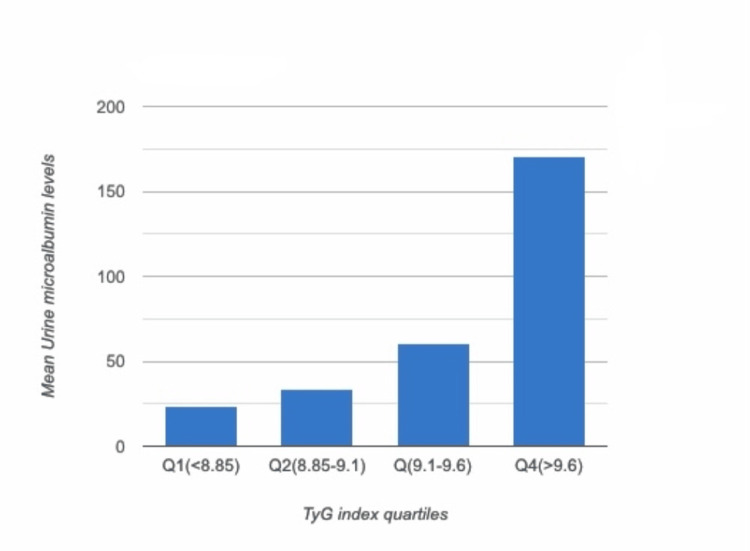
Severity of microalbuminuria across the TyG index quartiles TyG: triglyceride-glucose

**Figure 2:**
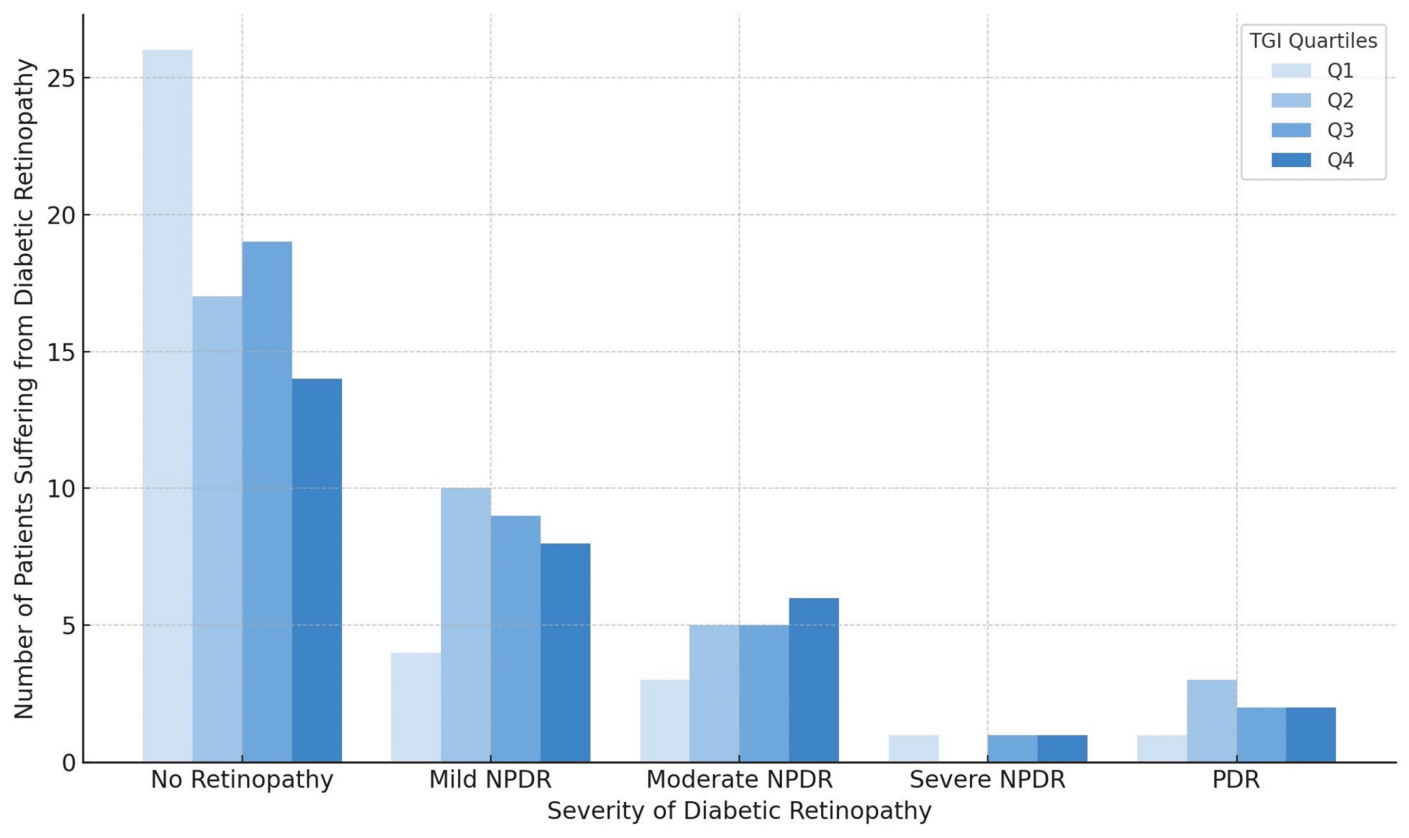
Distribution of patients by severity of diabetic retinopathy across TyG index quartiles TyG: triglyceride-glucose

**Figure 3:**
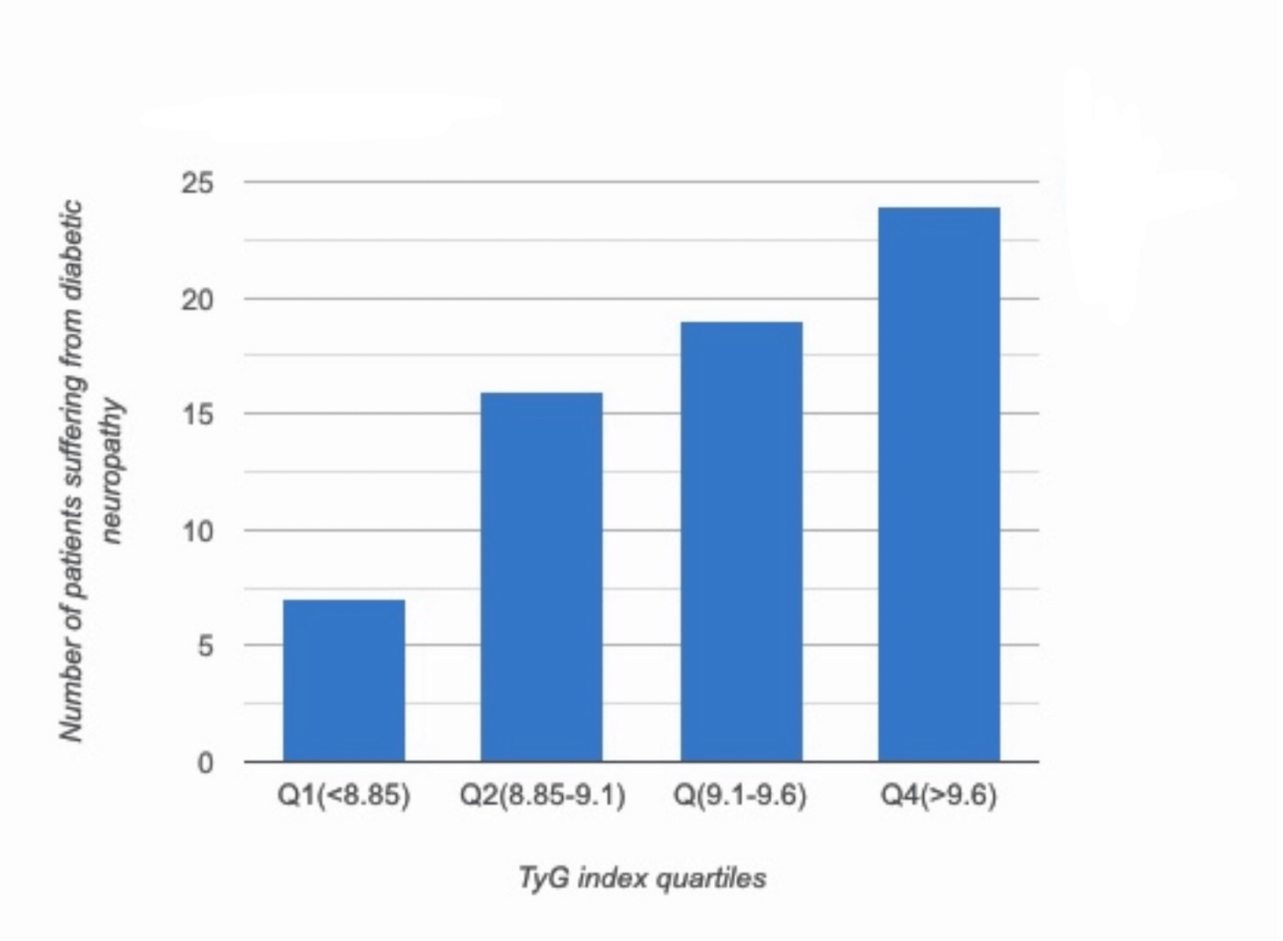
Patients suffering from diabetic neuropathy across the TyG index quartiles TyG: triglyceride-glucose

Of the 125 patients included in the study, 62 were found to have microalbuminuria, 51 were diagnosed with DR, and 66 had peripheral neuropathy. Chi-square analysis demonstrated that a higher TyG index was significantly associated (p < 0.005) with microvascular complications, including peripheral neuropathy and microalbuminuria, suggesting its potential role as a biomarker for metabolic dysfunction. Although DR showed a positive trend (p = 0.045), the association did not reach statistical significance, and no significant correlation was observed with macrovascular complications (p = 0.141). These findings highlight the relevance of the TyG index in predicting small vessel complications. This is further illustrated in three supporting graphs: Figure 1 depicting a progressive rise in mean urine microalbumin levels across TyG index quartiles, Figure 2 showing an increased number of patients with peripheral neuropathy, and Figure 3 demonstrating a shift toward more severe stages of DR with increasing TyG index values.

After checking for normal distribution using the Kolmogorov-Smirnov test (D = 0.0729 and p = 0.4975), stating a normal distribution of data, a way ANOVA test was carried out. It was noted that the severity of microalbuminuria varies with the four quantiles; the highest mean (170.328) was seen in quartile 4, while the lowest was seen in quartile 1. This parameter was statistically significant with a test value of 10.933* and a p-value of <0.001. Post hoc analysis showed that significant differences were seen between Q1 (<8.85) and Q4 (>9.6), Q2 (8.85-9.1) and Q4 (>9.6), and Q3 (9.1-9.6) and Q4 (>9.6) (Table 3). While at the same time, age, duration of diabetes, systolic blood pressure (SBP), and diastolic blood pressure (DBP) had no significant effect on TyG levels.

**Table 2:** Quantitative analysis of various parameters in the quartiles using one way ANOVA test TyG: triglyceride-glucose; ANOVA: analysis of variance; SBP: systolic blood pressure; DBP: diastolic blood pressure; DM: diabetes mellitus; BMI: body mass index

	Q1 (<8.85)	Q2 (8.85-9.1 )	Q3 (9.1-9.6)	Q4 (>9.6)	F/welch	p-value
Age	63.84 ± 12.707	63.19 ± 9.945	60.42 ± 11.075	57.59 ± 12.899	1.875	0.137
Duration of DM	7.5 ± 4.968	8.032 ± 4.167	7.839 ± 4.92	7.878 ± 6.315	0.059	0.981
BMI	25.572 ± 3.278	24.928 ± 3.561	25.591 ± 2.906	25.622 ± 3.853	0.296	0.828
SBP	131.29 ± 20.452	133.55 ± 19.757	127.42 ± 14.825	129.06 ± 16.917	0.674	0.57
DBP	83.23 ± 9.447	81.61 ± 8.601	80.97 ± 9.436	83.44 ± 10.957	0.492	0.689
Urine microalbumin (mg/dL)	23.168 ± 19.884	33.255 ± 25.354	60.761 ± 35.219	170.328 ± 301.365	10.933*	<0.001
Serum triglyceride	98.06 ± 18.285	113.03 ± 20.805	136.23 ± 19.561	212.06 ± 166.384	24.238*	<0.001
Fasting blood sugar	111.9 ± 17.009	151.9 ± 25.614	184.58 ± 34.426	244.94 ± 61.148	77.281*	<0.001
TyG index	8.584 ± 0.17	9.028 ± 0.106	9.413 ± 0.152	9.999 ± 0.405	190.689*	<0.001

**Table 3:** Post hoc analysis of one way ANOVA test studying microvascular complications of diabetes in the various TyG index quartiles TyG: triglyceride-glucose; ANOVA: analysis of variance; SBP: systolic blood pressure; DBP: diastolic blood pressure; DM: diabetes mellitus; BMI: body mass index

	Q1 (<8.85)-Q2 (8.85-9.1 ) (difference (p-value))	Q1 (<8.85)-Q3 (9.1-9.6 ) (difference (p-value))	Q1 (<8.85)-Q4 (>9.6 ) (difference (p-value))	Q2 (8.85- 9.1)-Q3 (9.1-9.6) (difference (p-value))	Q2 (8.85-9.1)-Q4 (>9.6) (difference (p-value))	Q3 (9.1-9.6 )-Q4 (>9.6) (difference (p-value))
Age	0.645 (0.996)	3.419 (0.661)	6.245 (0.155)	2.774 (0.788)	5.6 (0.236)	2.826 (0.775)
Duration of DM	-0.5322581 (0.977)	-0.3387097 (0.994)	-0.3776042 (0.991)	0.1935484 (0.999)	0.1546539 (0.999)	-0.0388945 (1)
BMI	0.6432258 (0.881)	-0.0193548 (1)	-0.0505746 (1)	-0.6625806 (0.871)	-0.6938004 (0.852)	-0.0312198 (1)
SBP	-2.258 (0.961)	3.871 (0.835)	2.228 (0.962)	6.129 (0.545)	4.486 (0.76)	-1.643 (0.984)
DBP	1.613 (0.913)	2.258 (0.794)	-0.212 (1)	0.645 (0.994)	-1.825 (0.877)	-2.47 (0.741)
Urine microalbumin (mg/dL)	-10.0871 (0.994)	-37.5935 (0.773)	-147.1604* (0.001)	-27.5065 (0.896)	-137.0733* (0.003)	-109.5668* (0.029)
Serum triglyceride	-14.968 (0.902)	-38.161 (0.303)	-113.998* (<0.001)	-23.194 (0.713)	-99.030* (<0.001)	-75.837* (0.004)
Fasting blood sugar	-40.000* (<0.001)	-72.677* (<0.001)	-133.034* (<0.001)	-32.677* (0.006)	-93.034* (<0.001)	-60.357* (<0.001)
TyG index	-.4445242* (<0.001)	-.8292265* (<0.001)	-1.4157343* (<0.001)	-.3847023* (<0.001)	-.9712100* (<0.001)	-.5865078* (<0.001)

An independent t-test was done to study the relationship between the TyG index and categorical variables such as DR (Table 4) and neuropathy (Table 5).

**Table 4:** Independent t-test studying the relationship between diabetic retinopathy and TyG index TyG: triglyceride-glucose; ANOVA: analysis of variance; SBP: systolic blood pressure; DBP: diastolic blood pressure; DM: diabetes mellitus; BMI: body mass index

Parameters	No retinopathy N = 74		Retinopathy present N = 51		t	p-value
	Mean ± sd			Mean ± sd		
Age	59.8 ± 12.26		63.31 ± 11.02		-1.641	0.103
Duration of DM	6.41 ± 4.8		9.84 ± 4.88		-3.899	<0.001
BMI	25.5 ± 3.32		25.33 ± 3.52		0.284	0.777
SBP	128.65 ± 19.11		132.75 ± 16.26		-1.25	0.214
DBP	82.84 ± 9.58		81.57 ± 9.67		0.725	0.47
Urine microalbumin (mg/dL)	46.96 ± 82.98		109.97 ± 232.16		-1.858	0.068
Serum triglyceride	138.5 ± 116.55		143.22 ± 53.68		-0.27	0.788
Fasting blood sugar	163.65 ± 61.49		188.78 ± 60.65		-2.259	0.026
TGI	9.16 ± 0.62		9.41 ± 0.47		-2.485	0.014

Duration of diabetes was also higher in those with retinopathy, with a t value of -3.899, and was statistically significant with a p-value of <0.001. Similarly, comparison of the TyG index between the two groups shows that the TyG index was higher in those with retinopathy, with a t value of -2.485, and was statistically significant with a p-value of 0.014.

**Table 5:** Independent t test studying the relationship between diabetic peripheral neuropathy and TyG index TyG: triglyceride-glucose; ANOVA: analysis of variance; SBP: systolic blood pressure; DBP: diastolic blood pressure; DM: diabetes mellitus; BMI: body mass index

Peripheral neuropathy	No (n = 59)	Yes (n = 66)	t	p-value
	Mean ± sd	Mean ± sd		
Age	59.47 ± 13.41	62.8 ± 10.11	-1.552	0.124
Duration of DM	5.74 ± 4.32	9.67 ± 5.06	-4.638	<0.001
BMI	26 ± 3.36	24.92 ± 3.36	1.789	0.076
SBP	130.68 ± 17.41	130 ± 18.73	0.209	0.835
DBP	82.37 ± 9.16	82.27 ± 10.05	0.058	0.954
Urine microalbumin (mg/dL)	31.59 ± 34.15	109.39 ± 217.02	-2.873	0.005
Serum triglyceride	123.68 ± 58.03	155.39 ± 118.3	-1.868	0.064
Fasting blood sugar	152.81 ± 46.85	192.76 ± 68.16	-3.851	<0.001
TyG index	9.04 ± 0.56	9.46 ± 0.52	-4.333	<0.001

Duration of diabetes was higher in patients with DR, with a t value of -4.638, and was statistically significant with a p-value of <0.001. Comparison of the urine microalbumin (mg/dL) between the two groups showed that urine microalbumin (mg/dL) was higher in those with diabetic neuropathy, with a t value of -2.873 and was statistically significant with a p-value of 0.005. Similarly, comparison of the TyG index between the two groups showed that the TyG index was higher in those having diabetic peripheral neuropathy, with a t value of -4.333 and was statistically significant with a p-value of <0.001.

Binary logistic regression was performed on the three groups of patients, i.e., DR, nephropathy, and neuropathy, to assess the association of demographic and clinical variables with the presence of microvascular complications of diabetes (coded as 1 = retinopathy/nephropathy/neuropathy present; 0 = no retinopathy/nephropathy/neuropathy). The model included age, gender, duration of diabetes, hypertension status, BMI, and triglyceride-glucose index (TGI) as predictors.

**Table 6:** Binary logistic regression studying the influence of various variables in diabetic retinopathy TyG: triglyceride-glucose; DM: diabetes mellitus; BMI: body mass index; df: degrees of freedom; SE: standard error; CI: confidence interval; OR: odds ratio

Variables in the equation	B	SE	Wald	df	p-value	Odds ratio	95% CI for OR lower limit	95% CI for OR upper limit
Age	0.018	.020	.862	1	.353	1.018	.980	1.059
Gender	-.549	.414	1.761	1	.185	.578	.257	1.299
Duration of DM	.145	.047	9.636	1	.002	1.156	1.055	1.266
Hypertension	.434	.409	1.131	1	.288	1.544	.693	3.440
BMI	-.004	.062	.004	1	.949	.996	.883	1.124
TyG index	1.096	.385	8.110	1	.004	2.992	1.407	6.363
Constant	-12.632	4.420	8.168	1	.004	.000	-	-

The overall model was statistically significant, and two predictors showed a significant association with the presence of microvascular complications of diabetes. Duration of diabetes was a significant predictor of DR (p = 0.002), with an odds ratio of 1.156 and a 95% confidence interval (CI) ranging from 1.055 to 1.266, indicating that for every one-year increase in the duration of diabetes, the odds of having retinopathy increased by 15.6%. The TyG index was also significantly associated with retinopathy (p = 0.004), with an odds ratio of 2.992 (95% CI: 1.407 to 6.363), suggesting that higher TGI values were associated with nearly a threefold increase in the odds of developing retinopathy. The logistic regression equation for predicting the log odds of retinopathy is as follows: 

$Logit\left( P \right)= -12.632+\left( 0.018\times Age \right)-\left( 0.549\times gender \right)+\left( 0.145\times\text{Duration of DM}\right)+\left( 0.434\times Hypertension \right)-\left( 0.004\times BMI \right)+\left( 1.096\times \text{TyG index} \right)$

**Table 7:** Binary logistic regression studying the influence of various variables in diabetic neuropathy TyG: triglyceride-glucose; DM: diabetes mellitus; BMI: body mass index; df: degrees of freedom; SE: standard error; CI: confidence interval; OR: odds ratio

Variables in the equation	B	SE	Wald	df	pvalue	Odds ratio	99% CI for OR lower limit	99% CI for OR upper limit
Age	.018	.021	.775	1	.379	1.019	.978	1.061
Gender	-.065	.453	.020	1	.887	.937	.386	2.279
Duration of DM	.222	.058	14.506	1	.000	1.248	1.114	1.399
Hypertension	-.492	.448	1.207	1	.272	.611	.254	1.471
BMI	-.124	.069	3.223	1	.073	.884	.772	1.011
TyG index	2.158	.514	17.621	1	.000	8.651	3.159	23.690
Constant	-19.231	5.339	12.974	1	.000	.000	-	-

**Table 8:** Binary logistic regression studying the influence of various variables in diabetic nephropathy TyG: triglyceride-glucose; DM: diabetes mellitus; BMI: body mass index; df: degrees of freedom; SE: standard error; CI: confidence interval; OR: odds ratio

Variables in the equation	B	SE	wald	df	p-value	Odds ratio	99% CI for OR lower limit	99% CI for OR upper limit
Age	.004	.022	.034	1	.853	1.004	.961	1.049
Gender	-.183	.458	.160	1	.689	.833	.339	2.044
Duration of DM	.110	.051	4.552	1	.033	1.116	1.009	1.235
Hypertension	.617	.449	1.886	1	.170	1.854	.768	4.474
BMI	.069	.068	1.035	1	.309	1.071	.938	1.223
TyG index	2.852	.559	26.079	1	.000	17.33	5.799	51.791
Constant	-29.420	6.122	23.093	1	.000	.000	-	-

In the same way, duration of diabetes and TyG index were consistently found to be significant predictors of diabetic neuropathy and nephropathy. For neuropathy, each additional year of diabetes was associated with a 24.8% increase in the odds (p = 0.000, OR = 1.248, 95% CI: 1.114-1.399), while higher TyG index values were linked to nearly an 8-fold increase in risk of developing diabetic neuropathy (p = 0.000, OR = 8.651, 95% CI: 3.159-23.690). Similarly, for nephropathy, diabetes duration was linked to an 11.6% increase in odds per year (p = 0.033, OR = 1.116, 95% CI: 1.009-1.235), and elevated TyG index levels showed a particularly strong association, with over a 17-fold increase in risk of developing diabetic nephropathy (p = 0.000, OR = 17.330, 95% CI: 5.799-51.791). These findings highlight the critical role of both duration of diabetes and TyG index in the development of microvascular complications. While other variables, including age (p = 0.353), gender (p = 0.185), hypertension status (p = 0.288), and BMI (p = 0.949), were not significantly associated with the outcome.

## Discussion

The TyG index offers a practical advantage in clinical settings, as triglyceride and glucose levels are commonly measured and incur minimal cost. Numerous studies have demonstrated its predictive value in individuals with metabolic disorders as well as in the general population. Nonetheless, there is a lack of focused clinical research examining whether the TyG index can predict the risk of future microvascular and macrovascular complications in patients with T2DM.

Of the 125 subjects included in the study, 62 were found to have diabetic nephropathy, 51 suffered from DR, and 66 had diabetic neuropathy. This study found a significant relation between the TyG index and all three microvascular complications of diabetes, an association that has been poorly studied in the literature. 

Diabetic nephropathy is a major healthcare challenge and occurs in about 0.9-62.3% of patients with diabetes in India, thus being a major cause of ESRD [19]. Diabetic nephropathy in its earliest stages presents with low levels of albumin in urine, known as microalbuminuria, thus being a reliable indicator for diabetic nephropathy. According to our study, the severity of microalbuminuria increased across the four quartiles, with the highest levels observed in the fourth quartile, indicating that the severity of microalbuminuria rises with a higher TyG index. Similar results were obtained by Pan et al. [20], where the TyG index was signiﬁcantly correlated with microalbuminuria but had no signiﬁcant correlation with CKD. This is in line with a previous study conducted by Liu et al., showing that the TyG index, as compared with HOMA2-IR, had a stronger correlation with urinary microalbuminuria but had no obvious correlation with estimated glomerular filtration rate (eGFR) [21]. These findings suggest that IR has a more pronounced effect on diabetic nephropathy in its early stages than in the later stages when renal function declines. Another study conducted by Chiu et al. [22] involving 1990 participants showed a positive correlation between microalbuminuria and the TyG index and significant trends in stepwise increases in albuminuria ≥ 30 mg/g corresponding to the quartiles of the TyG index. The Northern Shanghai Study [23] also found that a higher TyG index was associated with a higher risk of microalbuminuria; at the same time, this study also showed a significant correlation between the TyG index and CKD secondary to diabetic nephropathy in elderly Chinese community-dwelling individuals. Sreenivasan et al. [24], studying 1413 patients with T2DM, found out that albuminuria (defined as urinary albumin excretion ≥ 30 mg/24 hours) was associated with a higher TyG index, greater age, use of insulin, higher systolic BP, and the presence of DR. They also stated that albuminuria and DR are associated with each other. In a particular cohort study conducted by Rani et al., it was found that every sixth individual with T2DM has albuminuria and that they are twice as likely to have DR in the presence of microalbuminuria and six times as likely to have DR in the presence of macroalbuminuria [25].

While studying the patients with DR in our study, it was noted that patients with DR and a greater duration of disease had a higher TyG index and positively correlated with DR. While at the same time, those with severe nonproliferative diabetic retinopathy (NPDR) and proliferative diabetic retinopathy (PDR) were in the highest quartile of the TyG index, suggesting severe IR, and may offer some clue regarding the severity of DR. These findings were consistent with those carried out by Sreenivasan et al. [24]. We observed that fasting blood glucose levels increased progressively across higher quartiles of TyG index values. Given that persistent elevation of fasting blood glucose is strongly associated with the development of DR, this finding is particularly significant. Previous studies have demonstrated that the risk of DR increases markedly when fasting blood glucose exceeds 7.03 mmol/L and HbA1c levels rise above 6.4% (46 mmol/mol) [26]. These findings further reinforce the association between elevated fasting blood glucose, triglyceride levels, and the risk of DR. However, a study conducted by Chiu et al. [22] showed that there was no relationship between the TyG index and DR.

Most previous studies examining the relationship between the TyG index and microvascular complications of diabetes have not specifically assessed its correlation with diabetic neuropathy. Notably, Sreenivasan et al. [24] reported no significant association between the TyG index and diabetic neuropathy. In contrast, our study found a significant correlation between the two. Additionally, our analysis also revealed that patients with diabetic peripheral neuropathy had significantly longer diabetes duration, higher fasting blood sugar levels, elevated urine microalbumin levels, and a higher TyG index compared to those without neuropathy.

Our study did not show any positive correlation between the TyG index and macrovascular diabetic complications. This could be due to the small sample size and that only 44 out of 125 patients (35.2%) had macrovascular complications, which may be insufficient to detect a statistically significant association, especially when further divided among TyG quartiles. There might have also been underrepresentation of cases, as classification was based on history or existing diagnosis; hence, a clear picture could not be obtained.

Limitations

This study was conducted in a tertiary care center and may not be representative of the general population. Additionally, there is a significant gender imbalance, with a higher proportion of male participants, limiting the generalizability of the findings to both genders. As the study employed a cross-sectional design, exposure and outcome were measured simultaneously; therefore, temporal relationships could not be established. Furthermore, the study focused exclusively on diabetic patients and lacked a control group, which is a notable limitation. Furthermore, follow-up was not feasible, preventing an assessment of the prognostic significance of our findings and their association with the progression of microvascular complications and the TyG index. Lastly, potential confounding factors such as diet, exercise, smoking status, and medication adherence that can potentially affect both the TyG index and microvascular outcomes were not accounted for.

## Conclusions

This study underscores a significant association between the TyG index and the presence of microvascular complications in T2DM, namely, DR, diabetic nephropathy, and diabetic neuropathy. The TyG index, being a simple and cost-effective marker derived from routinely measured triglyceride and glucose levels, showed a strong correlation with urinary microalbumin levels, especially in the early stages of diabetic nephropathy. Additionally, patients with DR and neuropathy demonstrated higher TyG indices, with the most severe cases clustered in the highest TyG quartiles. These findings support the hypothesis that IR, as reflected by the TyG index, plays a critical role in the development and progression of microvascular complications. Notably, the observed trends align with findings from previous research, further validating the utility of the TyG index in clinical risk stratification for diabetic complications. Given the lack of extensive literature examining the TyG index in relation to all three major microvascular outcomes, our study contributes novel insights and emphasizes the need for larger, prospective studies to confirm these findings and explore the index’s predictive value over time.
